# Caregiver Mental Health, Quality of Life, and Coping Following a Child's Diagnosis of Autism: A Follow-Up Study After 4–6 Years

**DOI:** 10.1192/bjo.2024.272

**Published:** 2024-08-01

**Authors:** Chui Mae Wong, Sylvie Yiting Lian, Amily Yi Lin Ong, Lui Yee Ang, Iliana Magiati

**Affiliations:** 1 Department of Child Development, KK Women's and Children's Hospital, Singapore; 2 Duke-NUS Medical School, Singapore; 3 Yong Loo Lin School of Medicine, National University of Singapore, Singapore; 4 Lee Kong Chian School of Medicine, Singapore; 5 Department of Psychology, National University of Singapore, Singapore; 6 School of Psychological Science, University of Western Australia, Perth, Australia

## Abstract

**Aims:**

Caregivers of autistic children may experience greater stress and reduced mental well-being compared with caregivers of typically developing children or children with other neurodevelopmental conditions. Less is known about earlier child and family predictors of later caregiver stress, as most studies have been cross-sectional. This study aimed to examine how caregiver (coping strategies and appraisal of their child's autism) and child factors (behavioural difficulties and adaptive functioning) were related to mental health and quality of life in caregivers of 2–7-year-old autistic children over 4–6 years.

Methods: At Time 1 (T1), 119 caregivers completed the Coping Health Inventory for Parents (CHIP), Family Impact of Childhood Disability (FICD), Centre for Epidemiology Studies Depression Scale (CES-D), Autism Treatment Evaluation Checklist (ATEC), and Scales of Independent Behavior-Revised (SIB-R). Of those, 50 completed the same measures 4–6 years later (Time 2-T2). Demographic data at T1 and the World Health Organization Quality of Life (WHOQOL) questionnaire at T2 were also collected. The relative contributions of T1 caregiver and child factors in predicting T2 caregiver self-reported depression and quality of life were analysed with multiple regressions.

**Results:**

Caregivers’ depressive symptoms remained generally stable across 4–6 years (30% at T1 and 38% at T2 scoring at or above the CES-D cut-off), and earlier caregiver depression predicted later caregiver depression. At T2, child adaptive functioning significantly improved compared with T1, while mean child behavioural difficulties (e.g., behaviours disruptive to others, damaging to property, socially offensive or inappropriate) remained generally stable. Caregiver appraisal of the impact of child's autism on the family also did not change much over time, but higher T1 negative caregiver appraisals of their child's diagnosis predicted poorer later social quality of life on the WHOQOL. There were mixed findings regarding the helpfulness of coping patterns assessed by the CHIP, with our findings suggesting that family integration and optimism could be helpful in improving caregiver mental well-being.

**Conclusion:**

Modifiable predictors of longer-term caregiver adaptation indicate that in addition to providing early supports for children's adaptive functioning and social communication, caregivers’ appraisals of autism, caregiver and family coping strategies, and earlier caregiver depressive symptoms also need to be targeted.

